# Fitness-to-practice concerns in rural undergraduate medical education: a qualitative study

**DOI:** 10.1186/1472-6920-14-195

**Published:** 2014-09-19

**Authors:** Pamela Claire Snow, Pamela Jane Harvey, Kylie Lynette Cocking

**Affiliations:** School of Rural Health, Monash University, PO Box 666, Bendigo, Victoria 3552 Australia

**Keywords:** Fitness-to-practice, Medical education, Professional behaviour

## Abstract

**Background:**

Since July 2010, new reporting requirements have applied to registered Australian health practitioners who have a reasonable belief that a practitioner or student (of any registered discipline) is exhibiting “notifiable conduct”. A study of healthcare complaints reported that a small number of practitioners are over-represented in the majority of formal complaints brought against doctors. The impetus for conducting this research was a recognition that identifying and responding to particular behaviours early may prevent issues requiring mandatory reporting later on. As a first step, a better understanding of how fitness-to-practice (FTP) concerns are viewed was sought from stakeholders in a rural medical school.

**Methods:**

This qualitative project used purposive and snowballing sampling. Thirteen participants from an Australian rural medical school were interviewed for the study about FTP concerns. Seven were university staff, including clinical educators, program co-ordinators and academic faculty. Six were medical students in the middle of their final year. Their de-identified interview transcripts were independently coded into themes and emergent data categories were refined through comparative analysis between the authors. Data collection ceased after theoretical saturation was achieved.

**Results:**

Although students and faculty staff responded similarly in their recognition of FTP concerns, they varied in their assessment of their frequency, with students indicating that concerns were rare. Students and staff expressed reluctance to formally report students or colleagues with FTP concerns because of the complexity and uncertainty of medical practice. Both groups considered early recognition of problems and implementation of supportive mechanisms as important, but students generally did not want to contact the university about concerns for fear of stigmatisation.

**Conclusion:**

Education providers need to have clear processes for identifying and responding to FTP concerns in the pre-service years of medical training. Importantly, students need to feel that they can seek help for their own concerns and not be stigmatised in doing so. This is a difficult challenge in a profession that has a perceived culture of strength and a traditional hierarchy. Rural medical schools, with their smaller student groups, are well positioned for early response to issues of concern.

## Background

In 2006, the Productivity Commission delivered a report to the Australian Government recommending a single national registration board for health practitioners [1]. By the end of 2010, the establishment of the Australian Health Practitioner Regulation Agency (AHPRA) was complete and included the participation of all states and territories. Ten health professions were initially regulated, followed by a further four by the end of 2012 [2]. Student registration was launched in 2011, and there were 19,000 registered medical students in 2012 [2].

As of 1 July 2010, new reporting requirements now apply to registered health practitioners who have a reasonable belief that a practitioner or student (of any registered discipline) is exhibiting notifiable conduct (Table 1) [3]. National representative boards of health practitioner groups included in AHPRA created joint guidelines for mandatory reporting under the *Health Practitioner Regulation National Law Act* (known as the National Law). The guidelines are relevant to registered health practitioners of all professions, employers of practitioners and education providers. The National Law does not require students to make mandatory reports, but education providers must report students ‘…with an impairment that may place the public at substantial risk of harm’ [3] under section 143 of the act (Table 2).

**Table 1:** **National Law notifiable conduct**

**Section 140 of the National Law defines ‘notifiable conduct’ as where a practitioner has:**	a. Practised the practitioner’s profession while intoxicated by alcohol or drugs; or
	b. Engaged in sexual misconduct in connection with the practice of the practitioner’s profession; or
	c. Placed the public at risk of substantial harm in the practitioner’s practice of the profession because the practitioner has an impairment; or
	d. Placed the public at risk of harm because the practitioner has practised the profession in a way that constitutes a significant departure from accepted professional standards

**Table 2:** **National Law definition of student impairment**

**Section 5 of the National Law defines ‘impairment’ when the student:**	‘…has a physical or mental impairment, disability, condition or disorder (including substance abuse or dependence) that detrimentally affects or is likely to detrimentally affect the student’s capacity to undertake clinical training:
	a) As part of the approved program of study in which the student is enrolled; or
	b) Arranged by an education provider

The relatively new legislation in Australia on mandatory reporting is a response to local and global concerns about patient and public safety, best practice in health professions, and responding openly and fairly to issues of professional conduct concern. It has been reported that a small number of practitioners are over-represented in the majority of formal complaints brought against doctors, with 3% of registered doctors accounting for 50% of complaints [4]. High-risk practitioners appear to have behavioural patterns and ‘distinctive characteristics’ [5] that, if identified early in the complaint trajectory and remediated, may not persist and become notifiable behaviours. Bismark *et al*. report that medico-legal institutions, however, remain *reactive* to complaints rather than proactive about prevention [4].

There have been some studies focused on the behaviour of medical students during their pre-service education as a possible indication of subsequent notifiable behaviour [6–9]. Papadakis [8] *et al*. used a retrospective case-control methodology and reported that disciplinary action by a medical board was strongly associated with prior unprofessional behaviour in medical school, with particular concern about severe irresponsibility, severely diminished capacity for self-improvement, low scores on a Medical College Admission Test and poor grades in the first two years of medical school. Yates and James [10] identified undergraduate risk factors for professional misconduct that included male gender, early academic difficulties and, to a lesser extent, low socio-economic backgrounds.

The impetus for conducting this research project was a recognition that identifying and responding to particular behaviours early in a health practitioner’s educational and working trajectory may prevent issues requiring mandatory reporting and/or sanction later on. We had also been involved with a range of complex professional conduct issues prior to 2010 and recognised that the National Law requirements would have implications for how these issues will be managed in the future. In approaching this investigation of stakeholder views and experiences, we adopted the term ‘fitness-to-practise’ (FTP) based on Parker’s 2006 definition of a global competence that encompasses ‘clinical competence, acceptable professional behaviour and freedom from impairment’ [10]. It was recognised that this global competence definition reflects a continuum that ranges from low level concerns that are usually readily remediable, to extreme behavioural problems that encompass significant challenges for universities, at both policy and practice levels.

This study targeted students and staff in a rural/regional context because of the specific challenges associated with smaller residential populations in which community members often have overlapping social and work roles, making individuals less “anonymous” in a clinical education environment. It aimed to scope and understand stakeholder views on: a) the types of FTP issues and concerns encountered; b) how FTP issues and concerns are identified and defined in the pre-service medical student; c) what the response to FTP issues and concerns should be from educational providers and fellow students; and d) what effect the new legislative framework could have on decisions to report these issues.

## Methods

This qualitative study was based in an Australian government-funded Rural Clinical School (RCS) in a rural city with faculty staff and Year 5 (final year) student participants from the MBBS undergraduate program. The students had successfully completed Years 3 and 4 of the MBBS within the region. As the study was investigating stakeholder perceptions of fitness-to-practice behaviours, a qualitative methodology was chosen as most appropriate for the exploratory project design [11]. Approval for the study was granted by the University Human Research Ethics Committee.

### Participants

From fourteen invitations emailed to stakeholders, thirteen participants agreed to be interviewed for the study. Seven were university staff and included clinical educators, program co-ordinators and academic faculty. Six were medical students in the middle of their final year. The sampling was purposive, with PS and KC identifying university faculty and an initial selection of students for an invitation to participate. Attempts were made to purposively recruit a student who had displayed behavioural difficulties during the course of their studies, however this was unsuccessful. Student participation thereafter was based on snowballing, with participants asked to identify other students who might be interested in being involved. An initial explanatory statement was sent to identified staff and students, and consent for participation was sought after their expressions of interest were returned.

### Data collection

An interview protocol was developed after an initial literature review that included relevant AHPRA publications. Participants were interviewed by KC over a six month period either face-to-face (n = 9) or by telephone (n = 4, all students). The interviews were semi-structured, with general questions relating to key topic areas (Table 3), and were of about one hour in duration. Interviewees were not provided with copies of the interview probes beforehand, however the explanatory statement identified they types of issues to be covered. Each interview was audio-recorded and transcribed verbatim by KC.

**Table 3:** **Interview question protocol**

**Interview topic areas:**	● Can you tell me about your role in the MBBS program?
	● To what extent have you encountered these issues during your years as an educator/while you’ve been training?
	● How have you responded to such issues? What outcomes were you seeking/did you achieve? Did you consider the outcome(s) to be satisfactory?
	● What assistance were you able to enlist within your faculty/the university more widely?
	● How effective was this?
	● How important do you think it is that universities respond to FTP concerns in a proactive way during the pre-service years?
	● What other bodies/organisations have a role to play in addressing FTP concerns?
	● What factors have facilitated responses?
	● What have the barriers been?
	● Can you tell me about a particular occasion when you felt you achieved a good outcome?
	● What about an outcome that you were less happy with?

### Analysis

The de-identified interview transcripts were independently coded into themes by all authors to ensure reliability. Emergent data categories were refined through comparative analysis between the authors and collapsed after review into agreed final coding areas. The analysis used the principles of an emerging design of Grounded Theory that connects the coded categories to the emerging theory [11]. Data collection ceased after it was agreed that theoretical saturation was achieved.

## Results

Five main categories of response were identified:

- Specific FTP concerns and issues
- The extent to which FTP concerns are occurring
- Possible reasons why FTP concerns occur
- University staff and student responses to FTP concerns
- Attitudes towards mandatory reporting.

### FTP concerns and issues

Both university faculty staff and medical students noted issues around punctuality to class/clinic, talking about patients inappropriately, being ‘hung over’ in class and poor social behaviour outside the clinical placement. Faculty staff also spoke of concerns about immaturity, cultural differences with respect to communication etiquette, competency levels, personal hygiene and poor manners:

*‘I have certainly seen that in my years, students who actually sit in the canteen and talk about patients, I guess that’s unprofessional behavior, probably immaturity as well…’ (Staff 1)*

*‘…people laugh about the psych patients and you’re, like, this is not funny. If this were your brother or sister you’d be whistling a different tune’ (Student 2)*

*‘… it’s mainly been on their, their age, they are young, and they’ve been placed in a position of respect at a very early age and they can become a bit too personable with the patients…’ (Staff 5)*

Students mentioned a broad range of concerns, from illicit and prescribed drug abuse, to abuse of clinical record protocols, bullying, and sexism.

*‘…alcohol abuse…Usually binge drinking…There’s a reasonable amount of illicit drug use too…it really surprised me the amount of medical students that do, just because you know, it’s jeopardising your entire career’ (Student 1)*

Faculty staff and students were also concerned about student wellbeing and mental health issues:

*‘…depression and anxiety, um, sometimes it’s something more like um, you know, like bipolar or something, but not so often, um, and yeah so mostly just not coping and feeling overwhelmed and that can um, be you know, quickly overwhelming…’ (Staff 6)*

### Extent of FTP concerns and issues

Faculty staff and students varied in their assessment of the extent of FTP issues. Faculty staff responses ranged from *‘all the time’* to *‘very infrequent’*. The students, however, were clear in considering the rarity of severe FTP concerns and issues:

*‘I certainly haven’t, like, I haven’t seen anything specifically like, that I thought, oh that person isn’t fit to practice, but I do think that among medical students and particularly female medical students there’s quite a high level of anxiety…’ (Student 2)*

*‘I haven’t had a lot of issues in terms of the professional practice, I think it’s more the little things, not necessarily little, but things come up quite occasionally that can be a little bit concerning…’ (Student 4)*

### Reasons for FTP concerns and issues

Faculty staff expressed the view that FTP concerns among students related to their relative immaturity and young age, as well as inherent competition between students:

*‘…their behaviour is, you know, is twenty, twenty-one year old students, they’re doing what twenty or twenty-one year old students have done all their lives, exploring boundaries and finding, you know, their way in the world and they’ve got to make mistakes to learn, so you can’t be too critical of them, but you have to give them clear guidelines, but where those boundaries exist and where they do impact upon their ability to do what their profession expects of them, what their chosen profession expects of them…’ (Staff 2)*

FTP concerns after mid-year during the last year of the MBBS were considered a reaction to the lack of formal assessment in that cohort:

*‘…they already have an intern job so they don’t care’ (Staff 2).*

Student interviewees emphasised the stress of clinical and academic workloads, inadequate self-care, and some seeking to break away from the stereotype of the hard-working medical student.

*‘…you know one of the main problems I find with um students and practitioners is that they don’t look after themselves…as a student you pick up on, you learn from this by observing and noticing these things and it is really long hours and the work is difficult, so you need to step back a bit you know, and um, make a decision about work/life balance otherwise you can lose yourself and become subsumed by work’ (Student 6)*

*‘Medical students want to break the image of being a quiet nerd and I think if they go… again it’s the same reason they drink, it’s the over compensation again to kind of be, to separate themselves from that stereotype…’ (Student 1)*

### Responses to FTP concerns and issues

Both faculty staff and students considered early recognition of issues and implementation of supportive mechanisms as appropriate responses to FTP issues. Supervisory feedback, referral to appropriate medical and counselling services, and having opportunities for debriefing were regarded as important. Student welfare policies and guidelines were seen as essential for faculty staff to ensure access to appropriate remediation pathways:

*‘I think the earlier one can see there is a problem the better. From every point of view, um, the earlier in their training you can do it and um, and know who your high risk candidates are so you can find an appropriate way of dealing with them, rather than having to deal with it when it begins to declare itself and that’s often a] late and b] quite complicated by the time that occurs, and secondly; to create an atmosphere, and this is a very difficult thing to do, create an atmosphere that says we’re all human, that we all have vulnerabilities, that some people do better and worse in different circumstances, that our job here is to create rounded individuals um, and to help people in whatever way is essential…’ (Staff 3)*

*‘…it’s important to develop systemic responses to assist students, especially more so the ones who are struggling….ah, you need to be able to flag when a student is in difficulty… no one wants to ah, be seen to be ah,…not managing, for whatever reason, and so you have to um, you need to acknowledge human vulnerability and create an environment that ah, that seeks to help and support, instead of you know, punish or be punitive in ah, response, that doesn’t work, but we also need to encourage students, to ah, create the environments for them to admit problems, to see that that’s um, you know part of being professional too, knowing your limits and when you need help or support…’ (Staff 6)*

Students, however, reported that they were very unwilling to approach their university as a first-resort for assistance for fear of showing ‘weakness’:

*‘I think it’s really important for someone to intervene if someone at uni is having these problems as it can only escalate, but if I’m being perfectly honest I would never go to the uni for help, ever, ever, ever, ever, ever, ever purely because I just go to my family or I’d go see a GP or something.’ (Student 2)*

Faculty staff indicated that the small student numbers associated with the rural cohort was a distinct advantage in managing FTP issues and concerns early:

*‘I’d say having smaller student numbers here helps facilitate you know um, better management and identification of student problems and concerns and certainly inter-personal approaches you know, on the part of academics and support staff is crucial to assisting students with personal or other problems, I don’t think that’s always possible with larger groups of students and you can imagine they tend to fall through the gaps…’ (Staff 7)*

*‘…people need to feel that they matter actually, and how do you make, how do you create such an environment in classes of two, three, four hundred students?’ (Staff 3)*

### Reporting of FTP concerns and issues

Both faculty and students expressed reluctance to formally report colleagues or students with FTP concerns, and were generally unfamiliar with the AHPRA mandatory regulations. Unless obvious patient safety risks were at issue, faculty staff considered FTP concerns and issues as being subjective and mainly related to the uncertainty and complexity associated with medical practice. They generally felt there was a lack of intermediary remediation steps if colleagues were reported, for example:

*‘…the trouble is its all or nothing, there’s no accepted intermediate steps to address things and I mean we have had colleagues and we’ve all had people we know who have professional issues, but it’s all or nothing, there’s no step beyond reporting it to an authority, ah, most people feel very uncomfortable with that, because you know it might, it might just be personality conflict you know, how am I to judge whether one of my surgical colleagues complication rates is excessive or not?’ (Staff 2)*

Faculty staff also regarded it inappropriate to report students without trying other interventions first, for example:

*‘I’d be reluctant to report students where FTP issues come up, you know what is the context, are there other extenuating circumstances, is this a personal problem? Um, you know, I sit with a real awareness that students are away from home and often for the first time, um, and they’re learning new coping behaviours and having to accommodate a…a whole set of new stressors you know, like um, living away from home, um, study, new friends, managing money, socialising all those kind of things…’ (Staff 7)*

Students expressed extreme reluctance at reporting FTP issues for fear of being tracked as a “whistle blower”, destroying someone’s career or being wrong because of their lack of medical knowledge. They also mentioned the influence of the ‘hospital hierarchy’ and their position as students.

*‘I think it would hard for a medical student to say anything about another medical student, but I think it would be almost impossible to get a medical student to get the guts to say something about a medical doctor…’ (Student 2)*

*‘…it’s not possible to be perfect in such a highly complex area and I think most other, you know, practitioners are reluctant to ah, derail someone’s career over a mistake…one of the concerns I have with um, mandated reporting is that it can, you know, act as a disincentive to seeking help.’ (Student 6)*

Similarly, students reported that they were very reluctant to report any difficulties of their own for fear of stigmatisation:

*‘…I mean I’d really think twice about seeing, you know, a psychiatrist or um, if I had depression or something like that because it might ruin my career…’ (Student 6)*

## Discussion

There is a continuum of fitness-to-practice issues and concerns that range from low-level severity to those more extreme behaviours exhibited by a few ‘canny miscreants’ [12]. This study highlights that FTP concerns and issues among both faculty staff and students may be low level in terms of patient safety but high level when considering student (and future practitioner) wellbeing.

Although students report either experiencing or observing quite severe concerns about wellbeing, they are reluctant to seek help because of the distinct medical profession hierarchy that places students at the bottom, and not wanting to appear weak in a culture that values strength. This perception was reflected in the 2013 ‘National Mental Health Survey of Doctors and Medical Students’ [13] which investigated current stigmatising attitudes in the medical profession about practitioners with a history of mental health issues. Forty percent of the surveyed doctors and medical students agreed with the proposition that doctors were less competent if they had a history of mental health issues. Prevailing attitudes such as these create a difficult environment for student help-seeking behaviour, even though recent studies have shown that mental health issues affect undergraduate medical student progress [14]. Faculty staff, on the other hand, want to identify students with FTP concerns and issues as early as possible so that they can assist the student and also ensure that the university produces competent graduates. This dichotomy creates tension in the overall management systems designed to aid student welfare, however it is important to assist students to develop help-seeking skills during stressful times, including the transition from university to work [13].

On investigation, some FTP concerns will be identified as symptoms of underlying stress or anxiety in the student. Carefully eliciting the student perspective – in both practice and research projects – and responding appropriately is important for the best outcome. Many faculty participants commented on immaturity as a possible reason for FTP concerns and issues. As undergraduates, the student participants in this study were considered to be in late adolescence, a developmental stage significant in an individual’s continuing identity formation [15]. The implications of this and the fact that many mental health conditions have an initial emergent period during late adolescence [16] can collectively impact negatively on student wellbeing and engagement. The neurobiological development of executive functions such as self-regulation, planning and organisation also continues well into the mid-twenties [17]. The later maturation of certain areas of the brain, particularly the medial prefrontal cortex, has been implicated in the processing of risk and decision-making [18], perhaps contributing to FTP issues in some students.

The rural location of the MBBS program had both positive and negative implications. Smaller student cohorts enable faculty to monitor students more closely and assist with early identification and responses for students in need. Living, studying and working in smaller communities can create challenges around integration into local activities and managing dual roles as residents and professionals, particularly pertaining to professional boundaries [19].

Although this study was designed to better understand student needs and behaviour, there was some “blurring” of discussion topics in the interviews with students, with a number of student informants identifying behaviours of concern in some of the clinicians under whom they studied. The “mixed messages” this creates, and how students resolve such ambiguities should be a focus of future studies. There is also a suggestion in the data that students and practitioners are unfamiliar with the AHPRA regulations pertaining to mandatory reporting, and this should also be investigated further.

### Limitations

This study investigated a small sample of students and practitioners in an Australian regional setting and so may not be fully generalisable to other medical education contexts. It also looks at mandatory reporting through the lens of Australian legislation, which may differ in other jurisdictions. Although the interviews were conducted by KC, students were aware that PS is an educator in the program, which may have influenced decisions about what perspectives to share. Although efforts were made to recruit at least one student who had displayed professional behaviour difficulties, this was not successful, so we recognise that the sample is biased towards “non-problematic” respondents.

## Conclusion

Education providers need to have clear processes for identifying and responding to FTP concerns in the pre-service years of medical training. An early response to behavioural issues may assist in preventing the consolidation of undesirable attitudes and/or patterns of behaviour, or the development of the ‘distinctive characters’ that are evident in some practitioners exhibiting notifiable behaviour. Importantly, though, students need to feel that they can seek help for their own concerns and not be stigmatised in doing so. This is a difficult challenge in a profession that has a perceived culture of strength and a traditional hierarchy. Rural medical schools, with their smaller student groups, are well positioned for early response to issues of concern, assisting students in the development of appropriate professional formation. With the introduction of the new National Law on mandatory reporting, and increasing public interest in health professional regulation, it is likely that the future regulatory context will tighten. Universities, therefore, will need to have appropriate policies and practices in place to both minimise and ameliorate such issues.
